# Social Inequalities in T2DM-Related Risk Patterns and Diabetes-Related Knowledge Among Hungarian Secondary School Students Aged 16–20 Years: A Cross-Sectional Study Using an Adapted FINDRISC-Based Screening Framework

**DOI:** 10.3390/nu18081286

**Published:** 2026-04-18

**Authors:** Brigitta Füzesi, Gábor Ferenc Pörzse, Krisztina Antónia Bornemissza, Anita Horkai, Judit Sallai, Helga Judit Feith

**Affiliations:** 1 Faculty of Health Sciences, Doctoral School, Semmelweis University, 1085 Budapest, Hungary; fuzesi.brigitta@phd.semmelweis.hu; 2 National Federation of Diabetes Associations, 1116 Budapest, Hungary; 3 Department of Social Sciences, Faculty of Health Sciences, Semmelweis University, 1085 Budapest, Hungaryhorkai.anita@semmelweis.hu (A.H.); 4 Institute of Sociology, Faculty of Humanities and Social Sciences, Pázmány Péter Catholic University, 1088 Budapest, Hungary; 5 Central Department of Anaesthesiology and Intensive Care, North Pest Central Hospital—Honvéd Hospital, 1134 Budapest, Hungary; juditsallai@gmail.com

**Keywords:** FINDRISC, secondary school students, young people, socioeconomic status, diabetes-related knowledge, health behavior, type 2 diabetes

## Abstract

Background/Objectives: Type 2 diabetes mellitus (T2DM) is a major public health challenge, and several lifestyle-related factors associated with later T2DM may already emerge during the secondary school years. Socioeconomic status (SES), nutrition-related behaviors, physical activity, and basic diabetes-related knowledge may shape these early risk-related patterns. This study examined the relationships between SES, adapted FINDRISC-based T2DM-related risk patterns, and diabetes-related knowledge among Hungarian secondary school students aged 16–20 years. Methods: A nationwide, cross-sectional questionnaire survey was conducted among students attending Hungarian Baptist secondary schools (N = 1585). SES was classified by Two-Step cluster analysis based on parental education, parental occupation, number of books in the household, and frequency of travel abroad. Relative T2DM-related risk patterns were described using an age-adapted FINDRISC-based screening approach, and basic diabetes-related knowledge was assessed using a 12-item questionnaire. Associations were examined using cross-tabulation and regression analyses in SPSS version 27.0. Results: Most respondents fell into the lower categories of the adapted FINDRISC-based screening framework, whereas 7.4% were classified into the moderate or high adapted FINDRISC-based screening categories. SES was significantly associated with adapted FINDRISC-based screening categories (*p* < 0.001). Compared with the medium-SES group, students in the low-SES group had higher odds of belonging to a higher adapted FINDRISC-based screening category (OR = 1.81; 95% CI: 1.27–2.57; *p* = 0.001). SES was also significantly associated with basic diabetes-related knowledge profiles (*p* = 0.015); students with high SES were less likely to be in the low-knowledge group than in the high-knowledge group (OR = 0.62; *p* = 0.039). Conclusions: Social inequalities in T2DM-related risk patterns and diabetes-related knowledge are already visible among secondary school students aged 16–20 years. The adapted FINDRISC-based approach may be useful as a school-based, non-invasive descriptive screening framework for characterizing relative T2DM-related risk patterns, but it is not a validated risk prediction instrument for this age group.

## 1. Introduction

Non-communicable diseases (NCDs) are among the leading causes of mortality and morbidity worldwide, with type 2 diabetes representing an especially rapidly growing public health challenge [1]. In recent years, a growing body of research has highlighted that risk factors for diabetes may already be present during the teenage years, and that differences in health behaviors, particularly in nutrition and physical activity, can determine long-term health outcomes in adulthood [2,3].

Nutrition plays a key role in preventing T2DM, as confirmed by a recent systematic review. Evidence from studies conducted across European Union member states indicates that plant-based diets, whole grains, and regular consumption of fiber-rich foods are associated with a lower risk of developing T2DM. In contrast, the intake of ultra-processed foods, sugary drinks, and processed meats is associated with a higher risk [4]. Improving the quality of nutrition—particularly by increasing the consumption of whole grains—would be beneficial not only at the individual level but also from a health economic perspective, as supported by estimates from Finland [5]. These findings further justify examining dietary patterns when assessing early T2DM-related risk patterns in young people.

The likelihood of developing diabetes is influenced not only by biological or lifestyle factors but is also closely associated with socioeconomic status (SES) and cultural resources [6,7]. International research has consistently shown that obesity, physical inactivity, and unhealthy eating habits are more prevalent among young people with lower SES, all of which contribute to an increased risk of developing diabetes later in life [8,9]. International literature indicates that children and adolescents with lower SES generally have poorer diet quality, more unhealthy eating habits, and higher body mass index (BMI), which are closely linked to obesity and an increased risk of developing T2DM later in life [10]. A study examining dietary quality among Spanish adolescents found that poorer dietary quality is associated with lower SES, reduced physical activity, and other health-related factors [11].

Although numerous studies have documented the relationship between SES and unhealthy lifestyle behaviors among children and adolescents, few empirical, quantitative studies have specifically examined the relationship between SES background and risk factors for T2DM among adolescents [12]. An exception is a national study in Taiwan, which found that children and adolescents from low-income families had a significantly higher risk of developing T2DM compared to those from higher-income families [13]. Similarly, studies in Brazil have linked low SES to higher insulin resistance and unhealthy dietary patterns among adolescents, which may indirectly increase the risk of developing diabetes [14]. Recent review-level evidence supports this perspective, showing that children and adolescents with lower socioeconomic status are more likely to display less favorable health behaviors, including poorer diet quality and lower physical activity [15]. Studies likewise found a meaningful relationship between health literacy and adolescents’ health behaviors [16]. Together, these findings suggest that several behavioral and knowledge-related components of later adult-onset T2DM risk may already be socially patterned during the secondary school years. Available data on health behaviors of young people in Hungary indicate that social differences are reflected in lifestyle patterns and preventive health knowledge [17]. This focus is also relevant because the upper end of our sample overlaps with the emerging adulthood range. This developmental period remains underrepresented in diabetes prevention research despite its importance in linking childhood risk patterns with adult chronic disease prevention [18].

In multiple countries, questionnaire- and score-based tools are used to identify individuals with less favorable T2DM-related risk profiles, as they are inexpensive, non-invasive, and suitable for screening large populations. The best-known of these tools is FINDRISC (Finnish Diabetes Risk Score) [19], which was developed in Finland to estimate the 10-year risk of T2DM in adults. The widely used FINDRISC framework includes age, BMI, waist circumference, physical activity, vegetable and fruit consumption, history of elevated blood glucose or antihypertensive treatment, and family history of diabetes. Although glucose and oral glucose tolerance test data were available in the FINRISK surveys, these biochemical measures were not included among the final predictors of the score, because the aim was to develop a simple, non-invasive screening tool that could be applied without laboratory testing. This underlying logic is relevant to the present study, which used an adapted, questionnaire- and anthropometry-based framework to describe relative T2DM-related risk patterns among secondary school students aged 16–20 years rather than to estimate actual metabolic risk.

An adapted version of the FINDRISC questionnaire may provide a structured, non-invasive way to describe T2DM-related risk patterns in younger populations while accounting for lifestyle habits, body composition, and family history [19]. FINDRISC can be conceptually applied to younger school-based populations aged 16–20 years, as it is based on the same key risk dimensions of lifestyle, body composition, and family history. However, modification of the scoring is warranted because age—one of the strongest discriminative factors in the FINDRISC questionnaire (0 points under 45 years of age)—does not meaningfully contribute to risk differentiation in this age range. In this population, the overall screening profile is primarily shaped by factors related to body weight and health behaviors. Body composition, such as BMI and waist circumference, may warrant greater weighting, as these indicators are closely related to modifiable risk patterns associated with later T2DM risk at this age. Previous studies highlight the importance of including family history, as hereditary and ethnic factors carry greater relative weight in juvenile T2DM compared to adult-onset T2DM [20,21,22]. Therefore, international literature has tested rather than validated the tool in young populations. Jordanian university students (average age: ~20 years) completed the classic FINDRISC and could be categorized into low-, medium-, and high-risk groups [23]. However, the authors noted that the tool’s sensitivity was lower in younger populations due to age-related factors. These findings support the conceptual approach described above and indicate that the age scoring needs to be adjusted for use in this age group. A national survey in Indonesia used a modified FINDRISC and analyzed the younger age group (<35 years) separately. When a lower threshold was applied, the score proved to be an effective indicator of metabolic syndrome risk [24]. These findings support the notion that, with age-appropriate modification, the adult tool can be adapted for use in younger populations.

In other words, international examples do not support the existence of a FINDRISC version specifically validated for secondary school students aged 16–20 years. Still, they suggest that the underlying logic of FINDRISC can be extended to younger populations if age categories and thresholds are adjusted. However, these examples should be interpreted with caution, as most studies in younger populations have examined feasibility or screening utility rather than formal clinical validation against objective metabolic markers [25]. Therefore, in the absence of concurrent biomarkers such as HbA1c, fasting glucose, OGTT-based glucose values, or insulin resistance indices, the adapted FINDRISC should be understood as a preliminary, non-invasive screening framework rather than a validated risk prediction instrument for this age group.

In Hungary, a 2010 study screened school-aged children and adolescents for T2DM using risk factors (obesity, positive family history, and associated abnormalities) and identified those who required further evaluation [26]. The study followed the same logic as FINDRISC, developing a point-based risk score using readily available data. This approach is echoed by school-based screening in Hungary, conducted through a network of health visitors, in which students at higher risk were identified using a standardized questionnaire and anthropometry [27]. Together, these examples show that the principle of applying an adult-oriented risk logic to a younger population is also acceptable in Hungarian practice, even if the tool is not formally called FINDRISC.

The aim of this study was to examine the relationships between socioeconomic status (SES), adapted FINDRISC (Finnish Diabetes Risk Score)-based T2DM-related risk patterns, health-related behaviors, and diabetes-related knowledge among Hungarian secondary school students aged 16–20 years. More specifically, the study examined whether modifiable behavioral, family-related, and knowledge-related components of later adult-onset T2DM are already observable in this age group and whether these patterns are socially stratified. Rather than addressing clinically manifest diabetes in adolescence or emerging adulthood, our focus was on the early social patterning of risk-related behaviors and diabetes-related knowledge in a school-based population. Although the association between SES and T2DM has been extensively documented in adult populations, considerably less attention has been paid to adolescents and emerging adults. Early socioeconomic inequalities may influence health behaviors, lifestyle patterns, and metabolic risk factors long before the clinical onset of diabetes, making younger populations an important target for prevention-oriented research. In addition, most empirical evidence on SES-related inequalities in diabetes risk originates from Western Europe and North America, while relatively little research has examined these patterns in Central and Eastern European countries. This regional gap is particularly relevant given the pronounced socioeconomic health inequalities and the growing burden of metabolic diseases in this part of Europe. Hungary represents a notable case within this context due to its high prevalence of diabetes [28] and marked social gradients in health outcomes. Examining socioeconomic patterns of self-reported T2DM-related risk indicators among adolescents and emerging adults in Hungary may therefore contribute to a better understanding of early social determinants of metabolic health and provide evidence relevant for prevention strategies targeting younger populations.

Our findings may contribute to understanding how social background shapes prevention opportunities in this age group and may provide a basis for targeted school health promotion programs.

## 2. Materials and Methods

The study was conducted as a nationwide, cross-sectional, quantitative survey among students aged 16–20 years attending Baptist secondary schools in Hungary. The survey covered all Baptist secondary schools in the country and aimed to include the entire eligible student population within this school network. A total of 1697 questionnaires were returned, resulting in a response rate of 94.8%. Questionnaires with more than 25% missing answers were excluded from the analysis. Respondents younger than 16 or older than 20 were also excluded. A total of 112 questionnaires were excluded due to substantial missing data and/or age outside the predefined range. In total, 1585 valid questionnaires were obtained and included in the analyses. Missing values were treated as missing in the study and excluded from the relevant analysis.

While the study provides nationwide coverage of Baptist secondary schools, the findings should be interpreted with caution when generalizing to the entire population of Hungarian secondary school students.

This age range reflects the structure of the Hungarian educational system, in which upper-secondary schooling may include a language-preparatory year, school entry may be postponed by one year, and some school-based vocational pathways may extend students’ status beyond the usual age of secondary graduation. Participants younger than 16 years were excluded because their inclusion would have required additional consent procedures from a parent or legally authorized representative under the applicable Hungarian legal-ethical framework. Accordingly, the target population of the present study was defined as secondary school students aged 16–20 years rather than adolescents only.

This age range was selected not only for legal-ethical and school-system-related reasons, but also because late adolescence and emerging adulthood are developmental stages in which young people assume increasing responsibility for health-related lifestyle decisions, including eating habits and physical activity [29]. This age also represents a critical window during which changes in diet and physical activity can persist in later life. Therefore, focusing on students aged 16–20 years was considered methodologically appropriate, as this group is more likely than students below 16 years of age to participate actively in everyday decisions relevant to later T2DM-related risk, such as fruit and vegetable consumption, overall diet quality, and regular physical activity [30].

Data collection was voluntary and anonymous using a paper-based, self-administered questionnaire across 16 different schools. The questionnaire used in the study consisted of three main components that measured SES, risk factors, and diabetes-related knowledge. Data was processed in an aggregated, anonymized form to ensure full protection of participants’ identities.

Socioeconomic status of students was operationalized as a multidimensional construct reflecting cultural and economic capital, drawing on Bourdieu’s framework of capital. For this reason, SES was not represented by a single indicator but by a combination of parental education, parental occupation, number of books in the household, and frequency of travel abroad [31]. Parental education and parental occupation were included as conventional indicators of social position. At the same time, books in the household and travel abroad were treated as proxies for cultural capital and broader access to material and experiential resources. This multidimensional approach was considered more appropriate for a school-based sample aged 16–20 years than relying on income alone, because secondary school students may have limited or unreliable knowledge of household earnings, whereas these indicators are more observable and better suited to self-report in this age group. To identify internally similar yet socially distinct subgroups, Two-Step cluster analysis was applied. This method was chosen because it enables the joint use of multiple socioeconomic indicators and is well-suited to detecting naturally occurring respondent groups in large datasets. The resulting clusters were interpreted substantively based on their educational, occupational, and cultural-resource profiles, and were labeled as high-, medium-, and low-SES groups. This approach made it possible to examine whether diabetes-related risk patterns and diabetes-related knowledge differed across a broader constellation of social advantage and disadvantage, rather than single SES variables in isolation.

The original FINDRISC questionnaire is an eight-item, non-invasive adult screening tool that served as the conceptual basis for the present study [19]. Consistent with the original rationale of FINDRISC, which was designed to rely on questionnaire-based and anthropometric information rather than laboratory measures, the present adaptation was also conducted without biochemical markers and was intended as a school-based screening approach, not as a validated risk prediction instrument for this age group. In the present study, the scoring procedure was adapted for a school-based sample aged 16–20 years. Specifically, the age item was omitted from scoring because the original adult age categories do not meaningfully differentiate respondents within this relatively narrow age range. The study population comprised a relatively narrow age group, namely 16- to 20-year-olds; adolescents and young adults were analyzed together rather than as separate subgroups to maintain the stability of the findings. BMI was retained as a core indicator of body composition; however, classification was based on age-appropriate categories for participants aged 16–19 years, using the World Health Organization (WHO) BMI-for-age reference for 5–19 years [32]. Values below +1 SD were considered normal weight, values between +1 SD and +2 SD were considered overweight, and values above +2 SD were considered obese. For participants aged 20 years, adult BMI cut-offs were applied (<25 kg/m^2^; 25–29.9 kg/m^2^; ≥30 kg/m^2^), and scoring followed the original FINDRISC logic (0, 1, or 3 points). The remaining core domains of the instrument were retained, namely, waist circumference, physical activity, daily fruit and vegetable consumption, antihypertensive treatment, previously measured elevated blood glucose, and family history of diabetes. Physical activity was operationalized in an age-appropriate way using the frequency of intense physical activity lasting at least 60 min per day during the previous week. Waist circumference was classified using criteria applicable from age 16 onward, in line with the International Diabetes Federation (IDF) pediatric consensus [33], which permits the use of adult cut-offs from age 16 years. For Europid populations, these correspond to cut-offs of ≥94 cm for males and ≥80 cm for females. However, because universally accepted adolescent-specific waist-circumference thresholds are lacking [34], this component should be interpreted as part of an adapted risk-screening framework rather than a fully validated age-specific FINDRISC standard for 16–20-year-olds. The modified FINDRISC total score was calculated by summing the seven retained components and was computed only if valid data were available for at least six components. Participants were classified into five descriptive screening categories derived from the original FINDRISC framework: low (≤6 points), minimal (7–11 points), moderate (12–14 points), high (15–20 points), and very high (≥21 points). These category labels were retained solely for descriptive comparability with the original FINDRISC framework. Accordingly, the adapted score was used only to describe relative screening categories and should not be interpreted as a validated risk prediction instrument for 16–20-year-olds. No formal separate pretest of the adapted instrument was conducted prior to the main survey. Therefore, the adapted version should be interpreted as a theory-informed, school-based screening modification rather than as a pre-validated age-specific instrument.

Diabetes-related knowledge was assessed using a study-specific 12-item questionnaire developed for a general, non-clinical population of secondary school students aged 16–20 years. The questionnaire was conceptually informed by the Revised Diabetes Knowledge Test (DKT2) [35] and its Hungarian adaptation [36], both established instruments for assessing diabetes-related knowledge in clinical populations. However, because the present study was conducted among secondary school students without a diagnosis of diabetes, the original DKT2 was not used as an item-by-item instrument. Instead, the research team developed a brief, age-appropriate set of items focusing on foundational, publicly relevant knowledge domains, including the basic concept of diabetes, the role of insulin, the main types of diabetes, and selected lifestyle- and risk-related aspects of T2DM prevention. The questionnaire should therefore be interpreted as a content-informed measure of educational knowledge rather than a shortened, validated clinical scale. An overview of the modifications to the instruments used in the present study is provided in Table 1.

Data were processed using SPSS version 27.0, with multiple statistical procedures applied in sequence, each building on the results of the previous analysis. Descriptive statistics were used to summarize SES characteristics, including gender, age, place of residence, household size, parental education, and occupation. Two-Step cluster analysis was applied to group students by SES, using parental education, occupation, number of books in the household, and frequency of travel abroad as clustering variables. Cross-tabulation procedures were used to examine relationships among SES, diabetes knowledge, and lifestyle factors. Ordinal logistic regression was applied to analyze the association between SES and the adapted FINDRISC-based screening category, treated as an ordinal variable. Finally, multinomial logistic regression analysis was conducted to examine the association between SES and the level of knowledge regarding diabetes. This quantitative, cross-sectional, questionnaire-based design provided a basis for examining whether SES was already reflected in T2DM-related lifestyle, anthropometric, and family-history patterns, as well as in diabetes-related knowledge in this age group. The methodological approach allowed for the identification of associations between SES-related differences and health behaviors.

## 3. Results

Of the 1585 valid questionnaires, 1402 respondents had sufficient data to be included in the SES analysis, and 1236 of these were also evaluable for the adapted FINDRISC analysis. Diabetes-related knowledge was assessable in the full sample (N = 1585). The sample was balanced by gender (56.64% female, 43.36% male), with the majority of participants residing in urban areas. In terms of parental education, approximately one-third of the families had at least one parent with a higher education degree (father/stepfather: 35.10%; mother/stepmother: 43.20%), while a similar proportion had parents with a secondary education degree (father/stepfather: 29.60%; mother/stepmother: 33.55%). Approximately one-quarter of respondents reported that their fathers or stepfathers had vocational school or vocational secondary school qualifications, whereas this proportion is notably lower for mothers or stepmothers (16.41%). The proportion of respondents whose parents had attained no more than a primary school education is below 10% for both parents (7.63% for fathers/stepfathers and 6.83% for mothers/stepmothers).

Three SES groups were identified using Two-Step cluster analysis (Table 2). The high-SES group (37.38%) was characterized primarily by higher parental educational attainment and a more favorable overall cultural-resource profile. The medium-SES group (19.97%) included students with average educational attainment and heterogeneous occupational and cultural backgrounds. The low-SES group (42.65%) was characterized by lower parental educational attainment and a less favorable overall socioeconomic profile.

Relative T2DM-related risk patterns were described using an adapted version of the FINDRISC questionnaire. The majority of respondents fell into the lower categories of the adapted FINDRISC framework (70.15%), while 22.49% were classified into the minimal category and 7.36% into the moderate- or high-category groups. The most frequent less favorable components of the adapted FINDRISC framework were physical inactivity (36.35%), previously elevated blood glucose (16.2%), and family history of diabetes (47.33%) (Table 3).

There was a significant association between SES groups and adapted FINDRISC-based screening categories (*p* < 0.001; Cramer’s V = 0.119) (Table 4). The adapted FINDRISC framework distinguishes five descriptive screening categories (low, minimal, moderate, high, and very high). However, in the present sample, no respondents fell into the very high screening category; therefore, this category is not listed in Table 4. Accordingly, four categories (low, minimal, moderate, and high) are shown in the table.

The distributions show that respondents in the low-adapted FINDRISC-based screening category were predominantly from the high-SES group (40.44%). In contrast, individuals from the low-SES group were significantly overrepresented in the high-adapted FINDRISC-based screening category (81.82%). The medium-SES group exhibited a transitional pattern, with proportions across screening categories closely reflecting those of the total sample (Table 4).

Ordinal logistic regression was used to examine whether adapted FINDRISC-based screening categories differed across social status groups. A zero-order (simple, two-variable) model was analyzed, including a single predictor, the SES cluster variable, with no additional covariates. The results showed that the model was statistically significant (X^2^ = 22.73, *p* < 0.001), although the explained variance was relatively low (Nagelkerke R^2^ = 0.025). The comparatively low pseudo-R^2^ value may reflect the fact that the adapted FINDRISC-based screening categories are shaped by numerous behavioral, anthropometric, familial, and social factors. In contrast, our model examined only SES cluster membership. Among the social clusters, individuals in the low-social-status group were significantly more likely to be classified into a higher adapted FINDRISC-based screening category compared to the medium-status group (OR = 1.81, 95% CI: 1.27–2.57, *p* = 0.001), while no significant difference was observed between the high and medium SES groups (OR = 0.95, 95% CI: 0.65–1.38, *p* = 0.787).

Diabetes-related knowledge clusters were derived from 12 items, with response options simplified to facilitate the analysis (Table 5). Table 5 presents the item-level distribution of responses.

Cluster analysis was performed using 12 dichotomously coded diabetes knowledge items (0 = incorrect or “I do not know,” 1 = correct response), identifying three distinct knowledge profiles among respondents aged 16–20 years. The clusters were interpreted based on the average score for each item, with the averages representing the proportion of correct responses.

Cluster 1—Group with low and incomplete knowledge (n = 220)

This group is characterized by consistently low percentages of correct responses across the questions examined. The average number of correct responses is particularly low for basic biological concepts, such as the role of insulin (M = 0.18), its site of production (M = 0.15), and the different types of diabetes (M = 0.28). Performance also remained low for items related to lifestyle and prevention: only a small proportion of respondents correctly identified the risks associated with obesity (M = 0.22), the importance of regular physical activity (M = 0.22), and the influence of family history (M = 0.17). The predominance of incorrect and “I do not know” responses in this group indicates a substantial gap in foundational knowledge (Table 6).

Cluster 2—Group with moderate and selective knowledge (n = 499)

This group functioned as a transitional cluster between the other two groups and was characterized by a heterogeneous knowledge profile. For basic definitional items, the average number of correct responses was moderately high (e.g., definition of diabetes: M = 0.71; correct rejection of the statement “only adults can have diabetes”: M = 0.92). However, greater uncertainty was observed for more complex or less straightforward items. In this group, only a small proportion of respondents correctly identified the site of insulin production (M = 0.29), the preventability of T2DM (M = 0.36), and the risks associated with being overweight (M = 0.46). Overall, this group demonstrates predominantly selective knowledge: most students were familiar with basic, frequently emphasized concepts, yet substantial gaps remained in their understanding of more detailed biological and preventive factors (Table 6).

Cluster 3—Group with high knowledge (n = 866)

Students in this group answered nearly all questions correctly at a very high rate. They demonstrated strong knowledge of basic biological mechanisms. The majority correctly identified the definition of diabetes (M = 0.86), the main types of diabetes (M = 0.96), and the associations between obesity and sugar consumption (Overweight people are more likely to develop T2DM (M = 0.73); Eating too much sugar and other sweet foods and drinks can lead to being overweight (M = 0.94)). They also achieved high average scores in the lifestyle and prevention domains, including regular physical activity (M = 0.64) and awareness of family-related risk factors (M = 0.83). At the same time, some uncertainty was observed regarding more complex or less emphasized knowledge, as fewer respondents answered questions about the preventability of T2DM (M = 0.47) and the prevalence of type 1 diabetes (M = 0.15) correctly. This cluster, therefore, represents a group with a high, balanced, and stable level of knowledge, with incorrect or uncertain responses mainly associated with less intuitive or more technical knowledge (Table 6).

Based on multinomial logistic regression results, social status was significantly associated with the level of diabetes-related knowledge (X^2^ = 12.41, *p* = 0.015). Participants with high social status were less likely to belong to the low-knowledge group than to the high-knowledge group (OR = 0.62, *p* = 0.039).

## 4. Discussion

### 4.1. Lifestyle-Related and Anthropometric Risk Patterns Among Secondary School Students Aged 16–20 Years

The primary objective of this study was to describe lifestyle, anthropometric, and family history patterns associated with T2DM among Hungarian secondary school students aged 16–20 years, with particular emphasis on lifestyle habits and health conditions that may increase the risk of disease at a young age. Our results show that four-fifths of secondary school students aged 16–20 years were in the normal BMI range, while one-fifth were classified as overweight or obese according to the BMI categories used in the adapted FINDRISC framework. The proportion of respondents who consume vegetables and fruit daily (two-thirds) can be considered favorable; however, the remaining one-third exhibit serious deficiencies. Physical activity data are also noteworthy, as more than one-third of respondents exercised fewer than 2 days per week.

Although most respondents fell into the lower categories of the adapted FINDRISC framework, obesity, a sedentary lifestyle, and insufficient fruit and vegetable consumption in this age group reflect lifestyle behaviors that may be associated with an increased long-term risk of developing T2DM. These findings are consistent with international literature, which suggests that the early adoption of unhealthy lifestyles may contribute to metabolic abnormalities [2,3]. Obesity and poor-quality carbohydrate intake are strongly associated with the risk of developing T2DM, including in younger populations. Dietary patterns among secondary school students and young adults are largely shaped by family habits and choices, which are in turn strongly influenced by socioeconomic status, underscoring the importance of considering SES in this context [37]. Diet quality, including nutrient density and sustainability, is closely associated with the socioeconomic environment, which also affects dietary risks in this age group. This is further supported by a study of British adolescents, which found that a suboptimal diet significantly impacts macro- and micronutrient intake, potentially contributing to the risk of metabolic diseases, including T2DM [38].

Beyond socioeconomic status, nutrition, physical activity, body composition, and diabetes-related knowledge, mental health may also be relevant to school-age prevention. Previous studies suggest that chronic stress and depressive symptoms may be linked to adverse metabolic outcomes in young people, including central obesity and insulin resistance [39,40]. Although mental health was not assessed in the present study, school-based prevention programs may benefit from a broader biopsychosocial perspective that considers mental well-being alongside lifestyle-related diabetes risk factors.

### 4.2. The Role of Socioeconomic Background in Adapted FINDRISC-Based Screening Profiles

Our analysis of the adapted FINDRISC framework revealed statistically significant social differences in T2DM-related screening profiles. Members of the low-SES group had significantly higher odds of belonging to a higher FINDRISC-based screening category compared with those in the medium-SES group. The three SES clusters exhibited clearly distinct health patterns, and the observed associations closely reflected social gradients reported in international studies [41]. A clear relationship emerged between socioeconomic status and adapted FINDRISC-based screening categories: a more disadvantaged social position was associated with classification into less favorable adapted FINDRISC-based categories. The pattern shown in Table 4 is consistent with the possibility that social resources, such as parental education, cultural background, and access to lifestyle opportunities, are related to more favorable adapted FINDRISC-based screening profiles during the secondary school years. This finding is consistent with extensive research on social inequalities in adult populations, which indicates that lower SES is a major determinant of T2DM incidence [7,42].

This study also suggests that lower social status among secondary school students aged 16–20 years is associated with a less favorable self-reported profile of health behaviors and characteristics that may be linked to an increased long-term risk of T2DM. Social disadvantages were reflected in less favorable overall patterns of self-reported lifestyle- and risk-related characteristics, including physical activity, fruit and vegetable consumption, body-composition indicators, and previously elevated blood glucose. Consistent with previous studies, our findings indicate that social background is associated with self-reported T2DM-related screening patterns even before adulthood, with students from lower socioeconomic groups being more likely to exhibit a less favorable adapted FINDRISC-based screening profile [38].

### 4.3. Diabetes Knowledge and Social Differences

An important finding of the study is the presence of a clear social gradient in the 12-item basic diabetes-related knowledge questionnaire. Students from high-SES backgrounds performed better across all domains. Most students in the high-SES group correctly identified the basic mechanism of insulin function, whereas performance among low-SES students was more moderate, with nearly one-fifth selecting the “I do not know” response, indicating considerable uncertainty.

This knowledge gap is consistent with the literature on health literacy, which indicates that young people from lower socioeconomic backgrounds have less access to structured health information and are more likely to rely on inaccurate or informal sources [16,43]. The greater confidence in diabetes knowledge observed among higher-SES students may reflect both higher parental education and more favorable health behavior patterns. This is particularly relevant because international evidence suggests that diabetes-related knowledge itself may be associated with more favorable health behaviors relevant to prevention and may reduce engagement in health-risk behaviors [44].

The results indicate that socioeconomic background affects diabetes-related knowledge, with misconceptions and gaps in understanding its basic biological processes being more common among socially disadvantaged students. While the present cross-sectional findings should be interpreted with caution and do not permit causal inference, the coexistence of socially patterned differences in both basic diabetes-related knowledge and adapted FINDRISC-based screening profiles may nevertheless be relevant from a preventive perspective. This stage of education may be a suitable time for school-based health education, as it often overlaps with formal instruction in biology and chemistry, including topics such as nutrition, metabolism, and chronic disease prevention. In this context, schools may offer a feasible setting for strengthening basic diabetes-related knowledge while health attitudes and behavioral patterns are still evolving. The multinomial logistic regression analysis further confirmed that socioeconomic status was significantly associated with the level of basic diabetes-related knowledge; students from higher socioeconomic backgrounds were less likely to belong to the low-knowledge group. Importantly, we observed that knowledge gaps were not limited to students with low knowledge profiles. Even in the high-knowledge cluster, substantial uncertainty remained regarding the preventability of type 2 diabetes and other less intuitive basic diabetes-related concepts. This may suggest that basic diabetes-related knowledge in this age group is uneven rather than uniformly adequate, even among students with otherwise favorable knowledge profiles. These findings support the need for age-appropriate, school-based basic diabetes education in addition to lifestyle-oriented prevention [45]. Such an approach may be particularly important because this age is a critical period when health-related behavior patterns are established, and schools provide an important setting for strengthening health literacy, informed decision-making, and preventive action.

Taken together, the findings suggest that social inequalities in T2DM-related risk patterns are already observable among students aged 16–20 years and may reflect multiple, interrelated behavioral and knowledge-related differences. Students from less advantaged social backgrounds were more likely to fall into less favorable adapted FINDRISC-based screening categories and also showed less favorable lifestyle-related patterns and lower levels of basic diabetes-related knowledge. At the same time, the results indicate that basic diabetes-related knowledge in this age group is uneven rather than uniformly adequate. Even students with otherwise strong knowledge profiles showed uncertainty regarding important preventive aspects of the disease. This combination of social gradient, modifiable lifestyle-related factors, and incomplete preventive knowledge supports the relevance of considering integrated, school-based prevention strategies that combine screening, health education, and opportunities for healthier everyday choices.

### 4.4. Applicability of the Adapted FINDRISC-Based Screening Framework in a School-Based Population Aged 16–20 Years

FINDRISC was originally developed for adults, but several international studies have explored its use in younger populations [23,24]. A similar logic has been applied in Hungarian school-based screenings [25,26]. The results of the present study suggest that, with appropriate adjustments to age scales and threshold values, the adapted FINDRISC may be useful in this sample for describing relative screening patterns, organizing questionnaire- and anthropometry-based information into descriptive screening categories, and examining the social patterning of these categories. At the same time, because no objective metabolic markers were available in the present study, the tool should be interpreted as a preliminary, non-invasive screening approach rather than a validated risk prediction instrument for this age group. This nevertheless represents an important contribution to the Hungarian literature, as relatively few studies have quantitatively examined the relationship between T2DM-related screening patterns and social background in this population.

The findings also highlight the importance of family-level influences in shaping screening profiles for T2DM among secondary school students aged 16–20 years. Among the risk factors assessed by the adapted FINDRISC, family history emerged as a key component, suggesting that hereditary predisposition may contribute to higher scores within the adapted FINDRISC-based screening framework, even at relatively young ages. Together, family history and lifestyle-related factors may be relevant when considering early preventive approaches in this population. The explanatory power of the regression model was modest, unsurprising given that T2DM-related screening profiles are shaped by multiple behavioral, anthropometric, familial, and social factors, whereas our analysis focused only on socioeconomic background.

### 4.5. Strengths and Limitations

The strengths of this study include a nationwide coverage within the Hungarian Baptist secondary school network, an integrated examination of socioeconomic status, adapted FINDRISC-based screening profiles, and diabetes-related knowledge, and the use of an age-adapted FINDRISC instrument. The inclusion of students from secondary schools across the country and the relatively large sample size enhance the robustness of the analysis and provide broad coverage within the surveyed Baptist secondary school network.

Another important strength of the study lies in its multidimensional analytical framework. By simultaneously examining socioeconomic status, diabetes-related knowledge, and adapted FINDRISC-based screening profiles, the research provides a more comprehensive understanding of how structural social factors and individual-level knowledge interact to shape early health risks.

The study also benefits from the application of the FINDRISC questionnaire adapted for secondary school students aged 16–20 years. FINDRISC is a widely used and internationally recognized adult diabetes screening tool, and its use allowed the structured organization of non-invasive lifestyle-, anthropometric-, and family-history-related indicators into adapted screening categories. The age-adjusted adaptation of this instrument enabled the description of relative T2DM-related risk patterns in younger populations that are typically not included in standard diabetes screening frameworks.

Furthermore, by focusing on a relatively understudied population group, the study contributes to the growing body of research emphasizing the importance of early-life determinants of chronic disease risk. Adolescence represents a critical developmental stage during which health behaviors, dietary habits, and health literacy patterns are formed and consolidated. Understanding how socioeconomic background and diabetes-related knowledge are associated with adapted FINDRISC-based screening profiles in this age group may therefore provide valuable insights for early prevention strategies.

The primary limitation is the cross-sectional design, which precludes causal inference. Reliance on self-reported data may also have introduced bias in the reporting of lifestyle habits. Furthermore, because the sample was drawn from Baptist church-maintained secondary schools, the findings cannot be generalized to the entire Hungarian secondary school population. However, the survey had nationwide geographic coverage within the participating Baptist secondary school network. Therefore, the results may be regarded as broadly reflective of this surveyed school network.

Another limitation is that the adapted FINDRISC instrument was not formally pretested or psychometrically validated in a separate sample prior to its use in the present survey. Although the adaptation was based on the conceptual structure of the original FINDRISC and on age-appropriate modifications documented in the literature, future studies should examine item comprehension, feasibility, and criterion validity more systematically in the school-based population aged 16–20 years.

Accordingly, the adapted FINDRISC results should not be interpreted as reflecting actual metabolic risk or undiagnosed T2DM, but rather as self-reported, non-invasive screening patterns for T2DM in a school-based population.

Finally, a further limitation is that the 12-item diabetes-related knowledge questionnaire was developed specifically for this study and was not subjected to separate psychometric validation in an independent sample of secondary school students aged 16–20 years. Although the DKT2 and its Hungarian adaptation conceptually informed it, it should be interpreted as a brief educational knowledge measure rather than a formally validated short form of the original clinical instrument.

## 5. Conclusions

The present study yields three main conclusions. First, the original FINDRISC may serve as a conceptual basis for a school-based, non-invasive screening framework among secondary school students aged 16–20 years, provided that age-appropriate adaptations are made. The adapted tool may provide a useful descriptive framework for characterizing relative T2DM-related risk patterns and their socioeconomic distribution in this school-based population. However, in the absence of validation against objective metabolic markers, it should be interpreted as a descriptive screening framework rather than a validated risk prediction instrument for this age group. Second, socioeconomic background was associated not only with adapted FINDRISC-based screening categories but also with diabetes-related knowledge, and descriptive analyses further suggested less favorable lifestyle-related patterns among students with lower SES. Students with lower SES were more likely to show less favorable adapted FINDRISC-based screening profiles, lower levels of diabetes-related knowledge, and less favorable lifestyle-related patterns. Together, these findings suggest that social inequalities relevant to later health outcomes are already observable during the secondary school years. Third, basic diabetes-related knowledge in this age group was uneven and socially patterned. Although students with more favorable social backgrounds tended to perform better, important knowledge gaps remained even among students with otherwise favorable knowledge profiles, especially regarding prevention-related aspects of type 2 diabetes. This finding suggests that diabetes prevention during secondary school years may benefit from including not only lifestyle-oriented messages, but also basic, age-appropriate diabetes education.

Given the large sample size and the participants’ young age, these findings provide a useful empirical basis for refining ongoing T2DM prevention strategies in school settings. From a public health perspective, the results support considering early, school-based prevention approaches that do not rely solely on risk screening. Prevention efforts may benefit from combining the early identification of socially vulnerable students with age-appropriate diabetes education, practical nutrition education, and improved opportunities for regular physical activity. Such a combined approach may help reduce socially patterned health inequalities before they become further consolidated in adulthood.

Overall, the findings suggest that preventive strategies should address not only individual lifestyle factors but also the broader social context that shapes health opportunities for young people. Schools may therefore represent an important setting for promoting health equity and for supporting the early prevention of chronic diseases, including T2DM. Future studies should also examine younger age groups and additional contextual factors, such as mental well-being and school climate, to further refine prevention strategies for this age group.

## Figures and Tables

**Table 1:** Overview of modifications to the instruments used in the present study.

Instrument	Key Modification	Applied to Whom	Reason
FINDRISC	Age item omitted	All participants aged 16–20 years	The original adult age categories did not meaningfully differentiate respondents within this narrow age range
FINDRISC	BMI was classified according to the WHO BMI-for-age reference for participants aged 16–19 years, whereas participants aged 20 years were classified according to the original adult FINDRISC logic	Participants aged 16–19 years (WHO BMI-for-age classification); participants aged 20 years (adult FINDRISC classification)	To ensure age-appropriate interpretation of body-composition category while preserving comparability with the adult FINDRISC framework, where applicable
FINDRISC	Physical activity redefined in an age-appropriate way	All participants aged 16–20 years	To reflect activity patterns in a secondary school population using an age-appropriate indicator
FINDRISC	Waist circumference classified using criteria applicable from age 16 onward	All participants aged 16–20 years	Because the IDF pediatric consensus permits the use of adult cut-offs from age 16 onward in an adapted screening context
FINDRISC	Remaining core domains retained	All participants aged 16–20 years	To preserve the conceptual structure of FINDRISC while adapting the instrument for a school-based population
Study-specific diabetes-related knowledge questionnaire	Developed as a 12-item, age-appropriate questionnaire for a non-clinical secondary school student population aged 16–20 years	All participants aged 16–20 years	Because the study population was non-clinical and school-based, an age-appropriate educational knowledge measure was more suitable than a clinical diabetes knowledge instrument

**Table 2:** Standardized indicators of social status clusters (Two-Step cluster analysis) (N = 1402).

	Paternal Educational Attainment	Maternal Educational Attainment	Paternal Occupation	Maternal Occupation	Travel Abroad	Number of Books
High social status group (N = 524)	0.75	0.88	−0.17	−0.14	−0.37	0.57
Medium social status group (N = 280)	−0.06	0.06	0.78	0.19	−0.48	−0.17
Low social status group (N = 598)	−0.64	−0.79	−0.23	0.03	0.50	−0.39

blue = below average; white = sample average; red = above average. For the variable “travel abroad”, lower standardized values indicate more frequent travel abroad because the original response categories were reverse-coded.

**Table 3:** Distribution of adapted FINDRISC components among respondents aged 16–20 years (N = 1585, percentages are calculated based on valid responses for each FINDRISC component).

		Number of Respondents	Percentage
Waist circumference	men: <94 cm; women: <80 cm	879	60.54%
	men: 94–102 cm; women: 80–88 cm	454	31.27%
	men: >102 cm; women: >88 cm	119	8.20%
Have you ever had a high blood sugar level?	yes	251	16.18%
	no	1300	83.82%
Do you regularly take blood pressure medication?	yes	51	3.26%
	no	1513	96.74%
Do you eat vegetables and fruit every day?	yes	1040	66.58%
	no	522	33.42%
BMI category used in adapted FINDRISC scoring	<25 (normal weight)	1076	79.88%
	25–29.9 (overweight)	187	13.88%
	≥30 (obese)	84	6.24%
Diabetes—family history	yes, parent or sibling	175	11.15%
	yes, grandparent, aunt, uncle, or cousin	568	36.18%
	no	827	52.68%
Physical activity	≥3 days per week	993	63.65%
	≤2 days per week	567	36.35%

**Table 4:** Distribution of adapted FINDRISC-based screening categories by social status clusters (%).

Clusters	Adapted FINDRISC-Based Screening Category			
	Low (n = 867)	Minimal (n = 278)	Moderate (n = 67)	High (n = 24)
High social status group	40.44	29.96	35.59	13.64
Medium social status group	21.58	19.84	11.86	4.55
Low social status group	37.98	50.20	52.54	81.82
	100.0	100.0	100.0	100.0

**Table 5:** Distribution of responses to diabetes knowledge questions (N = 1585).

	Incorrect Response/ Did Not Know	Correct Response
A person becomes diabetic when their body cannot produce or use insulin.	23.09%	76.91%
Insulin is a hormone found in the blood that regulates blood sugar levels.	40.06%	59.94%
Insulin is produced by the pancreas.	54.13%	45.87%
Only adults can have diabetes.	17.67%	82.33%
There are two main types of diabetes: type 1 and type 2.	30.91%	69.09%
Eating too much sugar and other sweet foods and drinks can lead to being overweight.	18.55%	81.45%
Children with diabetes need to exercise regularly.	47.95%	52.05%
Type 2 diabetes can be prevented with a proper diet and exercise.	60.38%	39.62%
Overweight people are more likely to develop type 2 diabetes.	42.84%	57.16%
A family history of diabetes is a risk factor for developing diabetes.	37.29%	62.71%
Diabetes is a condition in which blood sugar levels are low.	49.78%	50.22%
Type 1 diabetes is the most common form of diabetes.	86.18%	13.82%

**Table 6:** Average scores for diabetes-related knowledge items across knowledge clusters (N = 1585).

	1. Group with Low and Incomplete Knowledge n = 220	2. Group with Moderate and Selective Knowledge n = 499	3. Group with High Knowledge n = 866
A person becomes diabetic when their body cannot produce or use insulin.	0.5227	0.7114	0.8649
Insulin is a hormone found in the blood that regulates blood sugar levels.	0.1773	0.6814	0.6594
Insulin is produced by the pancreas.	0.1500	0.2926	0.6328
Only adults can have diabetes.	0.1409	0.9178	0.9423
There are two main types of diabetes: type 1 and type 2.	0.2818	0.4048	0.9596
Eating too much sugar and other sweet foods and drinks can lead to being overweight.	0.0909	0.9078	0.9446
Children with diabetes need to exercise regularly.	0.2182	0.4449	0.6409
Type 2 diabetes can be prevented with a proper diet and exercise.	0.1909	0.3627	0.4677
Overweight people are more likely to develop type 2 diabetes.	0.2227	0.4549	0.7275
A family history of diabetes is a risk factor for developing diabetes.	0.1682	0.4709	0.8337
Diabetes is a condition in which blood sugar levels are low.	0.2000	0.3687	0.6559
Type 1 diabetes is the most common form of diabetes.	0.1955	0.0862	0.1536

## Data Availability

The raw data supporting the conclusions of this article will be made available by the authors on request. The data are not publicly available due to ethical reasons.

## Abbreviations

The following abbreviations are used in this manuscript:

BMI	body mass index
DKT2	Revised Diabetes Knowledge Test
FINDRISC	Finnish Diabetes Risk Score
IDF	International Diabetes Federation
NCDs	non-communicable diseases
SES	socioeconomic status
T2DM	type 2 diabetes
WHO	World Health Organization
